# Forced transport of self-propelled particles in a two-dimensional separate channel

**DOI:** 10.1038/srep24001

**Published:** 2016-04-01

**Authors:** Jian-chun Wu, Bao-quan Ai

**Affiliations:** 1 Guangdong Provincial Key Laboratory of Quantum Engineering and Quantum Materials, School of Physics and Telecommunication Engineering, South China Normal University, Guangzhou 510006, China

## Abstract

Transport of self-propelled particles in a two-dimensional (2D) separate channel is investigated in the presence of the combined forces. By applying an ac force, the particles will be trapped by the separate walls. A dc force produces the asymmetry of the system and induces the longitudinal directed transport. Due to the competition between self-propulsion and the combined external forces, the transport is sensitive to the self-propelled speed and the particle radius, thus one can separate the particles based on these properties.

Non-equilibrium transport in random environment has been an active field of research in theoretical and experimental physics. Especially, rectification transport in corrugated channels has received much attention and been extensively investigated in many processes from biology and chemistry to nanotechnology^1^,^2^. Usually, the corrugated channels fall into two categories: smoothly corrugated channels^3^,^4^,^5^,^6^,^7^ and compartmentalized channels^8^,^9^,^10^,^11^,^12^,^13^,^14^. Due to the confinement of separate walls, the transport in compartmentalized channels exhibits peculiar behaviors. For example, Hänggi and coworkers^13^,^14^ investigated the transport of an elongated particle in a 2D compartmentalized channel and found that the shape asymmetry of the particle can induce the occurrence of absolute negative mobility (ANM).

Recently, the study of self-propelled particles has attracted widely attention and shown lots of interesting new physics^15^,^16^,^17^. Self-propelled particles particles widely exist in nature and technology, and are assumed to have an internal propulsion mechanism, which can perform active Brownian motion by extracting energy from an external source. Under certain conditions, these particles could show interesting collective behaviors^18^,^19^,^20^,^21^,^22^ and spontaneous rectification transport^23^,^24^,^25^,^26^,^27^,^28^. For example, Wan and coworkers^23^ studied the rectification of overdamped swimming bacteria induced by an array of asymmetric barriers. Angelani *et al.*^24^ studied the run-and-tumble particles in periodic potentials and found that the asymmetric potential produces a net drift speed. Ghosh and coworkers^25^ studied the transport of Janus particles in a asymmetric channel and found that the rectification can be orders of magnitude stronger than that for ordinary thermal potential ratchets.

In studies on passive particles, some interesting phenomenons occur in well-designed channels in the presence of external driving forces^29^,^30^,^31^,^32^,^33^. Eichhorn and coworkers^29^,^30^ utilized a 2D periodic channel with tilted separate walls to trap particles exhibiting ANM, the appearance of ANM is mainly due to the diffusion of the driven particles depending on the channel geometry. Reguera *et al.*^32^,^33^ presented a particle separation scheme based on the combined action of external forces and entropic rectification, which induces the motion of particles of different sizes in opposite directions. The study of self-propelled particles driven by external forces have been also reported and may have some promising applications in nano-technology. For example, Costanzo and coworkers^34^ numerically investigated the separation of run-and-tumble particles with different velocities in microchannels, and found that the presence of a capillary flow affects the separation effect. In our recent publication^35^, a transversal ac force could influence significantly the transport of self-propelled particles in a 2D asymmetric period channel, and be applied to separate particles with different self-propelled speeds.

In this paper, we numerically investigate the transport of self-propelled particles moving in a 2D channel with perpendicular separate walls. We apply a tilted square wave force to trap the particle by the separate walls and add an constant force to produce the asymmetry of the system. These external forces mainly affect the diffusion and trap of the particles by the separate walls. The transport and its direction are determined by the competition between the mobilities along +*x* and −*x* directions. This work may stimulate some related experimental studies and potential applications such as particles separation.

## Model and Methods

**Figure 1:**
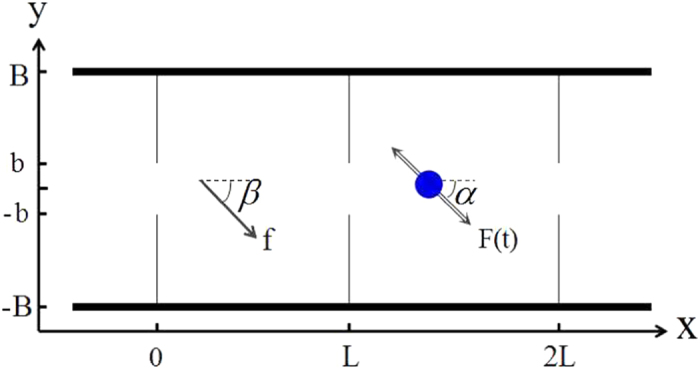
A spherical particle moving in a 2D channel with its periodicity *L*, width 2*B*, and bottleneck width 2*b*. The particle is driven by a square wave force *F*(*t*) with direction angle *α* and a constant force *f* with direction angle *β*.

We consider an self-propelled particle with the radius *R* moving in a 2D separate channel with period length *L*, width 2*B*, and bottleneck width 2*b* (shown in Fig. 1). The particle is driven by a square wave force 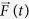 with direction angle *α* and a constant force 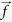 with direction angle *β*. By using the force 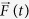, the particles can be trapped by the separate walls. However, in this time the system is symmetric. The added force 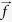 produce the asymmetry of this system, and the noises will play very important roles. The competition between the forces and the diffusion results in rich transport behaviors. In order to highlight the competition between the self-propelled force and the external driving force, we consider a dilute suspension and the particles moving in an overdamped regime, where particle-particle and particle-wall hydrodynamic interactions are neglected^19^,^20^,^36^,^37^,^38^. The dynamics of the particle can be described by the following Langevin equations^25^,


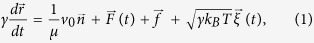



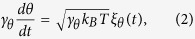


where 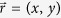 is the position of the particle. *μ* represents the mobility. *v*_0_ is the self-propelled speed, and the angle *θ* denotes its direction with respect to the channel axis. The unit vector 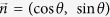. The translational diffusion *D*_0_ = *k*_*B*_*T*/*γ* with the Boltzmann constant *k*_*B*_, the temperature *T*, and the friction coefficient *γ*. The rotational diffusion *D*_*θ*_ = *k*_*B*_*T*/*γ*_*θ*_ with *γ*_*θ*_ being the rotational friction coefficient. 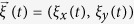 and *ξ*_*θ*_(*t*) model white Gaussian noise with zero mean and obey 〈*ξ*_*i*_(*t*)*ξ*_*j*_(*t*′)〉 = 2*δ*_*ij*_*δ*(*t* − *t*′), *i*, *j* = *x*, *y*, and 〈*ξ*_*θ*_(*t*)*ξ*_*θ*_(*t*′)〉 = 2*δ*(*t* − *t*′).

For a hard spherical particle with radius *R* moving in the channel, its geometrical shape is assumed not to change in the presence of external forces. By using the method^32^, the available space from the upper wall is described by the upper effective boundary


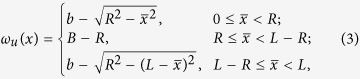


where 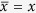 mod *L* is the modulo function. The lower effective boundary can be obtained by *ω*_*l*_(*x*) = −*ω*_*u*_(*x*).

For convenience, we introduce the dimensionless variables and choose the characteristic length *L* and energy *k*_*B*_*T*. Then the characteristic time *τ* = *L*^2^*γ*_*b*_/*k*_*B*_*T* with *γ*_*b*_ = 6*πνb*. Here *ν* is the shear viscosity of the fluid. Thus the friction coefficient of a particle of radius *R* is given by *γ* = *γ*_*b*_*R*_0_ with *R*_0_ = *R*/*b* being the scaled radius of the particle.

The Eqs (1) and (2) can be rewritten in dimensionless from^27^,


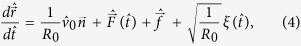



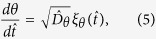


where 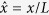, 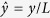, and 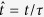. The rescaled parameters are 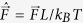, 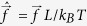, 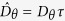, and 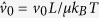. In the following, we only use the dimensionless variables and shall omit the hat for all quantities.

In this separate channel, it is very difficult to obtain the analytical expressions of the average velocity from the corresponding Fick-Jacobs equation. By considering the reflection boundaries, we solve Eqs (4) and (5) by using Brownian dynamic simulations with time step and the total integration time being 10^−5^ and 10^6^, respectively. Because the particle is confined along the *y* direction, we only calculate the average velocity along the *x* direction


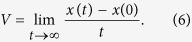


## Results and Discussion

**Figure 2:**
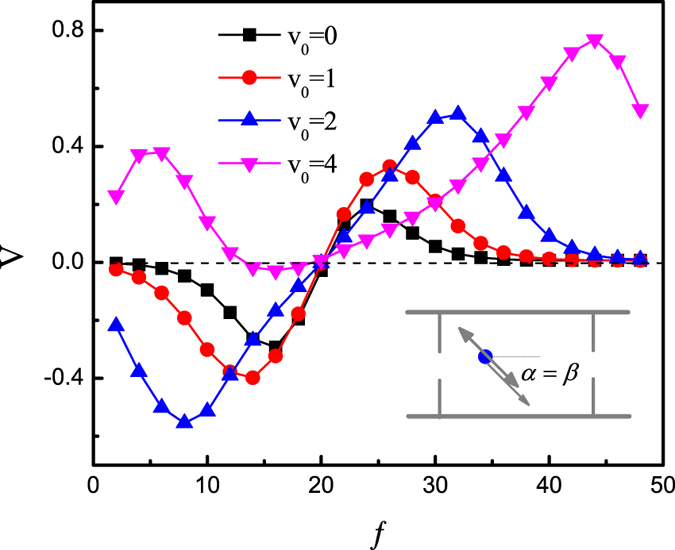
Average velocity *V* as a function of the constant force *f* at *F*_0_ = 20, *D*_*θ*_ = 0.1, *B* = 0.5*L*, *b* = 0.1*L*, *R*_0_ = 0.2, and *α* = *β* = −*π/*4 for different values of *v*_0_.

First of all, we consider the case *α* = *β* = −*π*/4 shown in Fig. 2. We plot the average velocity *V* as a function of the constant force *f* for different values of *v*_0_. When *f* → 0, the system is absolutely symmetric, the average velocity tends to zero. When *f* → *F*_0_ = |*F*(*t*)|, the particle will diffuse freely or be trapped by the lower separate walls, the average velocity tends to zero. When *f* is very large, the particle will be trapped tightly by the lower separate walls, thus the average velocity goes to zero. In Fig. 2, the transport and its direction both change with the increasing of *f*. These results comes from the competition of the particle mobilities along +*x* and −*x* directions produced by the constant force *f*. For self-propelled particles, the effective diffusion 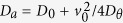 with the thermal diffusion *D*_0_ = *k*_*B*_*T*/*γ*, which increases significantly with *v*_0_^39^. When *v*_0_ = 4.0, a small constant force can obviously enhance the +*x* direction mobility, thus the average velocity is positive.

Obviously, the tilted constant force *f* produce the asymmetry of the system. We can divide *f* into two parts: a longitudinal component force *f*_*x*_ and a transversal component force *f*_*y*_. The force *f*_*x*_ induces normal particle mobility, while the force *f*_*y*_ mainly affects the particle diffusion and its trap. In the same way, we can divide *F*(*t*) into a longitudinal component force *F*_*x*_(*t*) and a transversal component force *F*_*y*_(*t*) for simplifying the following discussions.

**Figure 3:**
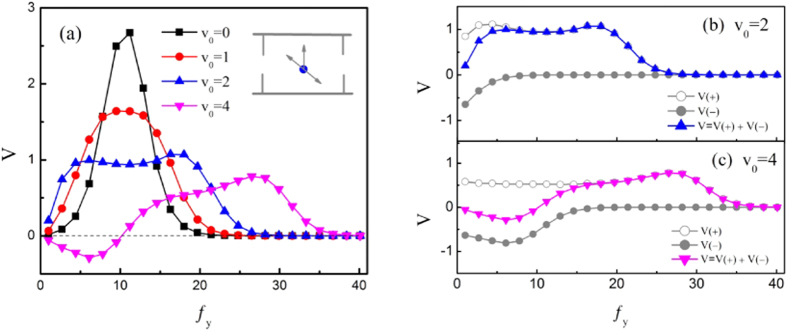
(**a**) Average velocity *V* as a function of the force *f*_*y*_ at *F*_0_ = 20, *D*_*θ*_ = 0.1, *B* = 0.5*L*, *b* = 0.1*L*, *R*_0_ = 0.2, and *α* = −*π*/6 for different values of *v*_0_. (**b**,**c**) plot the average velocities *V*(+) and *V*(−) versus *f*_*y*_ at *v*_0_ = 2 and *v*_0_ = 4, respectively.

Here, we take the force *f* along the transversal direction, i.e., *f*_*x*_ = 0 and *f*_*y*_ ≠ 0. In Fig. 3(a), we displays the average velocity *V* as a function of *f*_*y*_ for different values of *v*_0_. For a passive particle (i.e., *v*_0_ = 0), the average velocity *V* exhibits a nonmonotonic behavior with the appearance of a peak. There is an optimized value of *f*_*y*_ ≈ *F*_*y*_ at which the average velocity takes its maximal value. With the increasing of *v*_0_, the particle diffusion increases and its mobility will vary obviously with the constant force *f*_*y*_. In Fig. 3(b,c), the average velocities *V*(+) and *V*(−) represent the half of the steady state average velocity corresponding to the case of *F*_*x*_ > 0 and *F*_*x*_ < 0, respectively. From these figures, it is found that the transversal forces can facilitate the mobility of self-propelled particles. When *v*_0_ = 2, the average velocity *V*(+) takes its maximum at the total transversal force |*f*_*y*_ + *F*_*y*_| = 7 with *F*_*y*_ = −10. Thus there are two optimized values of *f*_*y*_ (*f*_*y*_ ≈ 3 and 17) at which the average velocity *V*(+) take two maximums. The average velocity |*V*(−)| decreases monotonically with *f*_*y*_, thus the two maximums of *V* = *V*(+) + *V*(−) are not equal, see the blue line in Fig. 3(b). When *v*_0_ = 4, the average velocities *V*(−) and *V*(+) take their maximums at the total transversal force |*f*_*y*_ + *F*_*y*_| = 16.5 with |*F*_*y*_| = 10. The average velocities *V*(−) and *V*(+) can take their maximal values at *f*_*y*_ ≈ 6.5 and 26.5, respectively. Thus the average velocity *V* can take a negative maximum and a positive maximum, see the magenta line in Fig. 3(c).

**Figure 4:**
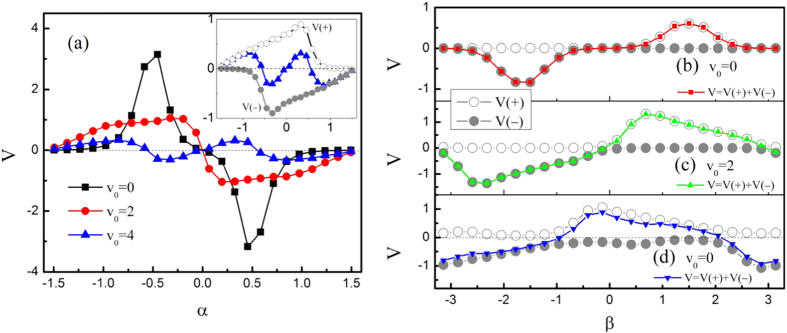
(**a**) Average velocity *V* as a function of the angle *α* at *β* = *π*/2 for different values of *v*_0_. Inset: *V*(+) and *V*(−) versus *α* at *v*_0_ = 4. (**b**–**d**) plot the average velocities *V*(+), *V*(−), and *V* versus *β* at *α* = −*π*/4 for *v*_0_ = 0, *v*_0_ = 2, and *v*_0_ = 4, respectively. Other parameters are *F*_0_ = 20, *f* = 10, *D*_*θ*_ = 0.1, *B* = 0.5*L*, *b* = 0.1*L*, *R*_0_ = 0.2.

Figure 4(a) plots the average velocity *V* as a function of *α* for different values of *v*_0_ at *β* = *π*/2. When *α* = 0 and *α* = ±*π*/2 ≈ ±1.57, the present system is symmetric along the channel axis, thus the average velocity is zero. From this figure, the average velocity exhibits very complicated behavior. Here we only analyse the case of *v*_0_ = 4 in detail in the inset. It is found that *V*(+) first increases, and then decreases with the increasing of *α* from −*π*/2 to *π*/2. From Fig. 3(c), the maximum of particle mobility occur in the case of |*F*_0_sin *α* + *f* | = 16.5, thus the optimal *α* = 0.33 for *V*(+) and *α* = −0.33 for *V*(−). The average velocity *V* is the sum of *V*(+) and *V*(−), and the direction of the transport can be reversed many times. These results are in agreement with our analysis. By utilizing the same way in Fig. 4(a), one can analyse the results in Fig. 4(b–d). The transport exhibits complicated behavior and is affected significantly by the angle *β*.

**Figure 5:**
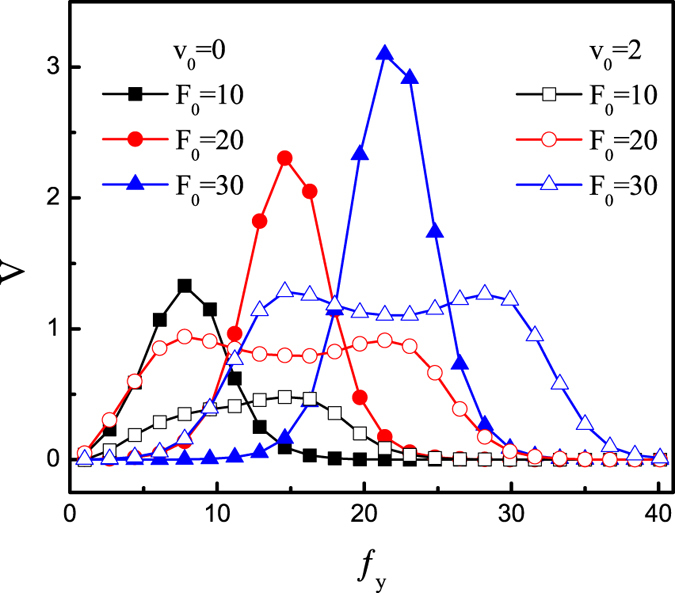
Average velocity *V* as a function of the force *f*_*y*_ for different values of *F*_0_ at *D*_*θ*_ = 0.1, *B* = 0.5*L*, *b* = 0.1*L*, *R*_0_ = 0.2, and *α* = −*π/*4.

Figure 5 plots the average velocity *V* as a function of *f*_*y*_ for different values of *F*_0_ at *α* = −*π*/4. The average velocity *V* can take its maximum at *f*_*y*_ = *F*_*y*_ for *v*_0_ = 0 and may take two maximums for *v*_0_ = 2, similar results have been shown in Fig. 3(a). As *F*_0_ increases, the maximal values of *V* increase and the corresponding optimized *f*_*y*_ shift to larger values. Thus one can improve the rectification transport by increasing the square wave force.

**Figure 6:**
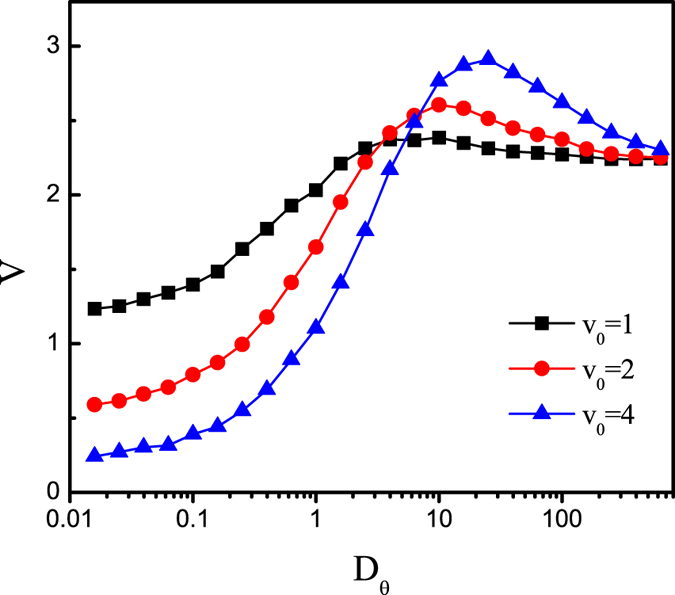
Average velocity *V* as a function of the rotational diffusion *D*_*θ*_ for different values of *v*_0_ at *F*_0_ = 20, *f*_*y*_ = 10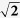, *B* = 0.5*L*, *b* = 0.1*L*, *R*_0_ = 0.2, and *α* = −*π*/4.

The dependence of *V* on the rotational diffusion *D*_*θ*_ is presented in Fig. 6. As *D*_*θ*_ increases, the average velocity *V* first increases, and then decreases. There exists an optimal value of *D*_*θ*_ at which the average velocity is maximal. When *D*_*θ*_ is very large, the active particle will reduce to a passive particle and the average velocity approaches to a fixed value.

In nature and technology, many systems are mixtures of different species. In the following, we will present particles separation based on the self-propelled speed and particle size.

**Figure 7:**
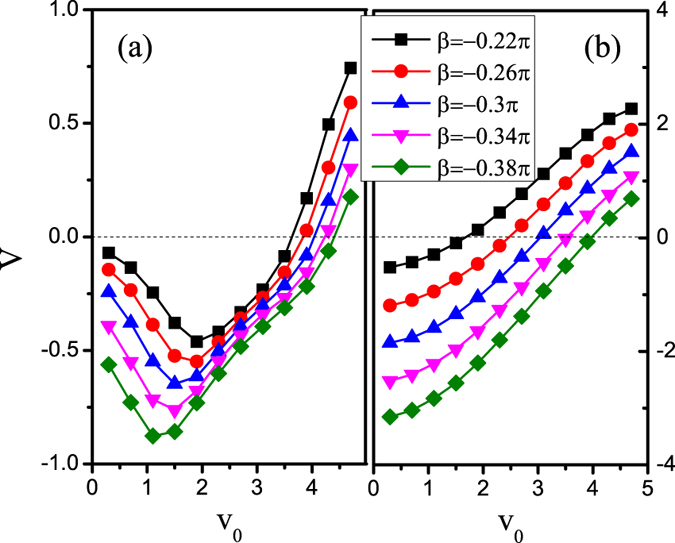
(**a**) Average velocity *V* versus *v*_0_ at *B* = 0.5*L*. (**b**) Average velocity *V* versus *v*_0_ at *B* = 0.2*L*. Other parameters are *F*_0_ = 20, *f* = 10, *D*_*θ*_ = 0.1, *b* = 0.1*L*, *R*_0_ = 0.2, and *α* = −*π*/4.

In Fig. 7(a), we plot the average velocity *V* versus the self-propelled speed *v*_0_ at *B* = 0.5*L*. Self-propelled particles smaller than a threshold self-propelled speed move to the left, whereas particles larger than that move to the right. Due to the separate walls are very long, the transport direction is not sensitive to the driving forces. Thus the controlled threshold self-propelled speed only changes in a small region by varying the values of *β*. If the separate walls is short, the transport direction may be more easier to adjust by varying the driving forces. Figure 7(b) describes that the average velocity *V* versus the self-propelled speed *v*_0_ at *B* = 0.2*L*. It is found that the average velocity increases monotonically and the controlled threshold self-propelled speed can adjusts by varying the values of *β*, which may be more convenient and effective than the previous method^35^.

**Figure 8:**
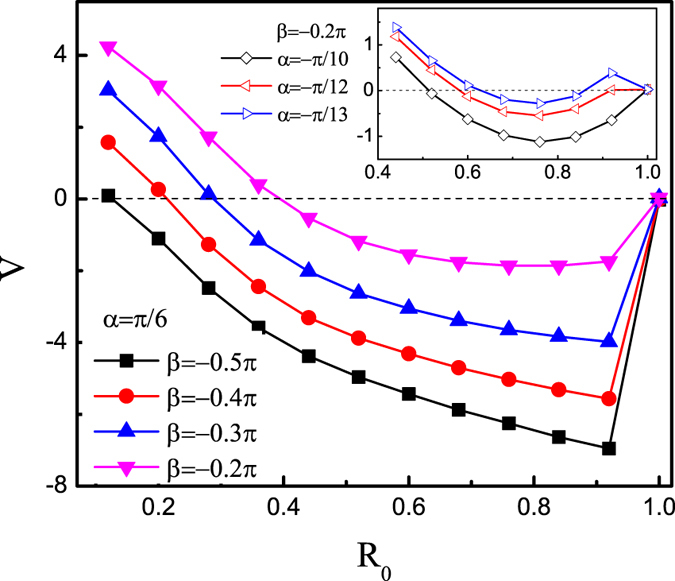
Average velocity *V* versus *R*_0_ for different values of *β* at *α* = −*π*/6. Inset: Average velocity *V* versus *R*_0_ at *β* = −0.2*π*. Other parameters are *F*_0_ = 20, *f* = 10, *D*_*θ*_ = 0.1, *B* = 0.2*L*, *b* = 0.1*L*, and *v*_0_ = 4.

Particle size is another factor affecting their diffusion and mobility. We plot the average velocity *V* as a function of the particle radius *R*_0_ in Fig. 8. By adjusting the direction angle *β*, particles smaller than a threshold radius move to the right, while particles larger than that move to the left. However, this way is not very useful for separating particles with large radius. In the inset, we display the average velocity *V* versus the particle radius *R*_0_ at *β* = −0.2*π* by varying the direction angle *α*. When *α* = −*π*/13, the transport direction can reverse several times, thus one can obtain particles with specific radius.

**Figure 9:**
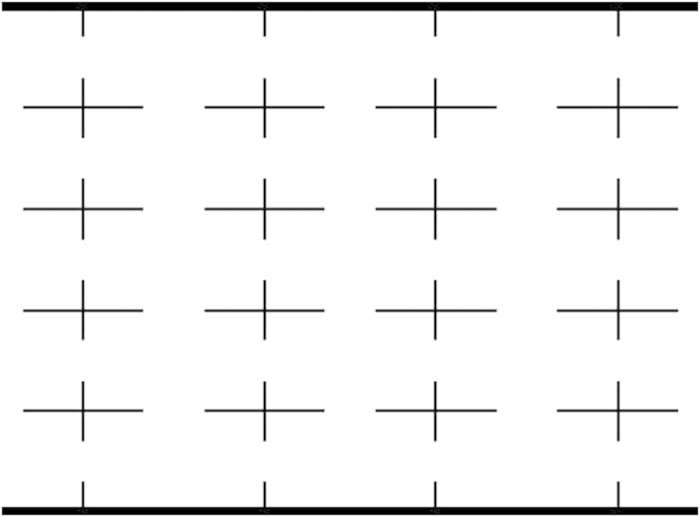
A extension of the channel from Fig. 1 for improving the efficiency of particle separation.

The results in this paper suggest a method to control the transport of self-propelled particles. In real experiments and industrial applications, improving their efficiencies are very important. In Fig. 9, we present an immediate geometrical modification of the channel from Fig. 1. The addition of separate walls along *y* direction can transport more particles, thus enhance the efficiency of particle separation.

## Concluding Remarks

In conclusion, we numerically investigate the transport of the spherical self-propelled particles in a 2D separate channel in the presence of the combined external forces. The tilted square wave force and the tilted constant force can strongly affect the particle diffusion and trap. The particle mobilities are sensitively dependent on the strengths and directions of the external forces. The transport and its direction can be controlled by these parameters (*v*_0_, *α*, *β*, *D*_*θ*_, *B*, and *R*_0_), which result from the competition between the mobilities along +*x* and −*x* directions. There exists an optimized value of the rotational diffusion at which the transport takes its maximal value. The diffusion of self-propelled particles depends on the self-propelled speed. The particles with different self-propelled speeds move in the opposite directions by adjusting the direction angle of the constant force. Because particles size can influence the diffusion and mobility, we also present a separation method based on the particles radius by adjusting the direction angles of external driving forces.

In this work, we only choose some particular cases to study the transport versus the related parameters. However, the results will not change substantially by choosing different direction angles of the driving forces and the self-propelled speeds. Our studies contribute to further understand particle transport mechanism in confining structure, and suggest a very effective method for controlling the particle transport in realistic applications.

## Additional Information

**How to cite this article**: Wu, J.-c. and Ai, B.-q. Forced transport of self-propelled particles in a two-dimensional separate channel. *Sci. Rep.* **6**, 24001; doi: 10.1038/srep24001 (2016).
